# The proto-oncogene survivin splice variant 2B is induced by PDGF and leads to cell proliferation in rheumatoid arthritis fibroblast-like synoviocytes

**DOI:** 10.1038/srep09795

**Published:** 2015-05-22

**Authors:** Sho Mokuda, Tatsuhiko Miyazaki, Yuki Ito, Satoshi Yamasaki, Hiroko Inoue, Yun Guo, Weng-Sheng Kong, Masamoto Kanno, Kiyoshi Takasugi, Eiji Sugiyama, Junya Masumoto

**Affiliations:** 1 Department of Immunology, Graduate School of Biomedical and Health Sciences, Hiroshima University, Hiroshima, Japan; 2 Department of Internal Medicine, Center for Rheumatic Diseases, Dohgo Spa Hospital, Matsuyama, Japan; 3 Department of Pathology, Ehime University Proteo-Science Centre and Graduate School of Medicine, Toon, Japan; 4 Department of Clinical Immunology and Rheumatology, Hiroshima University Hospital, Hiroshima, Japan

## Abstract

Survivin is an independent prognostic factor for joint destruction in rheumatoid arthritis (RA). However, the expression and function of survivin in RA synoviocytes remain unclear. We certified the expression of survivin in RA synovial tissues and performed the experiment using RA fibroblast-like synoviocytes (RA-FLS) treated with siRNA. As a result, the expression levels of wild type (WT) survivin and the 2B splice variants in RA synovial tissues were higher than those in osteoarthritis tissue samples, and, these variants were highly expressed in RA-FLS. The expression levels of survivin-WT and -2B in the RA-FLS were upregulated by PDGF. Treatment with siRNA against survivin-2B led to decreased viability of PDGF-treated RA-FLS due to cell cycle suppression and apoptosis promotion, while the siRNA against all survivin isoforms did not affect the viability. Moreover, an overexpression of survivin-2B in RA-FLS led to cell proliferation through cell cycle activation and by conferring resistance to apoptosis. In conclusion, survivin-2B has an important role in RA-FLS proliferation. These data suggest that survivin-2B might contribute to rheumatoid synovial hyperplasia, and have the potential as a novel therapeutic target for RA.

Rheumatoid arthritis (RA) is a chronic inflammatory disease characterized by hyperplastic synovial tissue and destruction of articular cartilage and adjacent bone^1^. The proliferative synovial tissues from RA patients consist of mainly of synoviocytes, capillary vessels and infiltrated lymphocytes. The cartilage destruction is caused by matrix metalloproteinase, which is produced by fibroblast-like synoviocytes (FLS)^2^. The bone destruction (bone erosion) in RA results from osteoclast-mediated bone resorption activated by RANKL, produced by FLS and T lymphocytes^3^,^4^. Therefore, FLS are major effector cells involved in RA synovitis. Moreover, RA-FLS exhibit pre-neoplastic characteristics. Several reports have indicated that RA synovitis might be characterized by elevated expression of proto-oncogene proteins in the FLS (for example, c-Myc, Ras, c-fos and p53 mutants)^5^,^6^,^7^,^8^,^9^,^10^.

Proto-oncogene survivin is a member of the IAP (inhibitor-of-apoptosis) family of proteins. It is encoded by the *BIRC5* gene located on human chromosome 17q25, and is a 142 amino acid, 16.5** **kDa, protein, which contains a single BIR (baculovirus *iap* repeat) domain and a coiled-coil α-helix domain^11^. Survivin is overexpressed in various cancers, and has been suggested to be involved in cancer development, progression and resistance to treatment^11^. Most normal differentiated cells do not express this protein, while other IAP family proteins (xIAP, cIAP1 and cIAP2) are comparatively ubiquitously expressed^12^. Therefore, survivin has attracted attention as a target for cancer therapy^13^.

In 2005, it has been reported that the survivin protein and antibodies against survivin were measurable in blood and synovial fluid from RA patients^14^. In addition, they also reported that the serum survivin level was capable of predicting the joint destruction in early RA^15^. And, other authors noted that the origin of survivin detected in blood or synovial fluid from RA patients was synovial tissues^16^. However, the expression of survivin in the RA synovial tissues has been controversial^16^,^17^,^18^. For example, real-time PCR analyses showed that the survivin mRNA expression levels in RA and osteoarthritis (OA) synovia were similar^17^,^18^.

In the present study, we evaluated the expression pattern of survivin and its splice variants in RA synovial tissues and compared them to osteoarthritis (OA) tissues, and examined whether survivin might be involved in pathological RA-FLS proliferation using small interfering RNA (siRNA)-mediated knockdown and *in vitro*-transcribed (IVT) mRNA transfection.

## Results

### The survivin expression in sera and synovium of RA and OA patients

**Figure 1:**
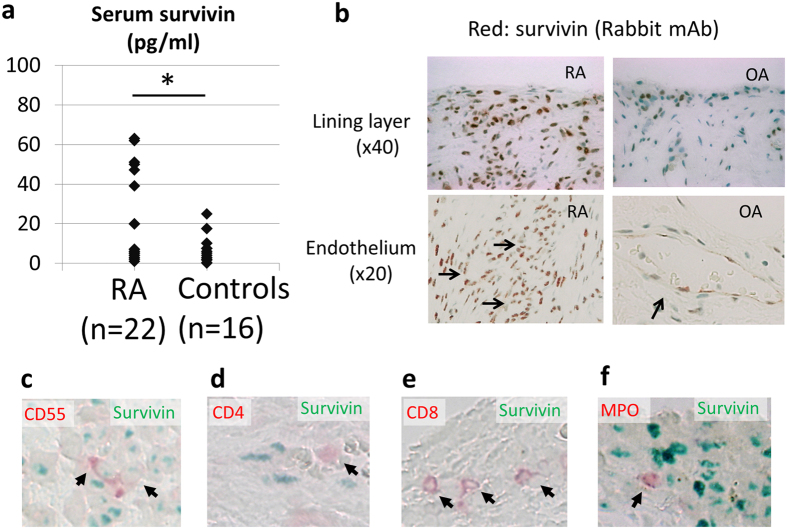
The survivin expression in blood and synovial tissues from RA and OA patients. (**a**) The serum survivin expression levels were analyzed using an ELISA. (RA, 22 cases; controls, 16 cases) (mean RA, 19.5** **pg/ml vs. controls, 7.7 pg/ml; p = 0.044). An age- and sex-matched control group consisted of OA and healthy controls (RA, 58.8 ± 15.6 years old; controls, 53.9 ± 19.8 years old; p = 0.404). t-test, *p < 0.05. (**b**) The survivin expressions in RA and OA synovial tissues were analyzed using IHC (survivin: red, upper sections x40, lower sections x20). The data shown are representative of the samples. Arrows indicate the endothelium of synovia. (**c**–**f**) Dual staining in immunohistochemistry for RA synovial tissues. Red pigment and arrows indicate the expression of each lineage marker (CD55; a marker of FLS, CD4; a marker of CD4^+^ T lymphocytes, CD8; a marker of CD8^+^ T lymphocytes, MPO (myeloperoxidase); a marker of neutrophil). Green pigment indicates survivin. (x40)

To clarify the survivin expression in RA patients and controls, the expression levels in sera and synovial tissues were investigated. As shown in Fig. 1a and Supplemental Table 1, the serum survivin levels in RA patients were higher than those in controls. In addition, survivin was expressed in rheumatoid synovial tissues, mainly at the site of synoviocytes (the intimal lining layer) and vascular endothelial cells (Fig. 1b). Survivin was also detected in OA synovial tissues, although the expression levels were lower than those in RA patients (Fig. 1b). The RA synovial tissues mainly consisted of synoviocytes and infiltrating leukocytes. It has been reported that some normal adult tissues also express survivin, including the synovial tissue, T lymphocytes and polymorphonuclear neutrophils^16^,^19^,^20^. To identify the characteristics of survivin expressing cells, we performed dual staining via immunohistochemistry (IHC) for synovial tissues (Fig. 1c–f). We observed that CD55 (a marker of FLS)-positive cells expressed survivin intracellularly. However, we could not detect survivin expression in most of the infiltrating T lymphocytes and neutrophils.

### Survivin splice variants are expressed in synovial tissues

**Figure 2:**
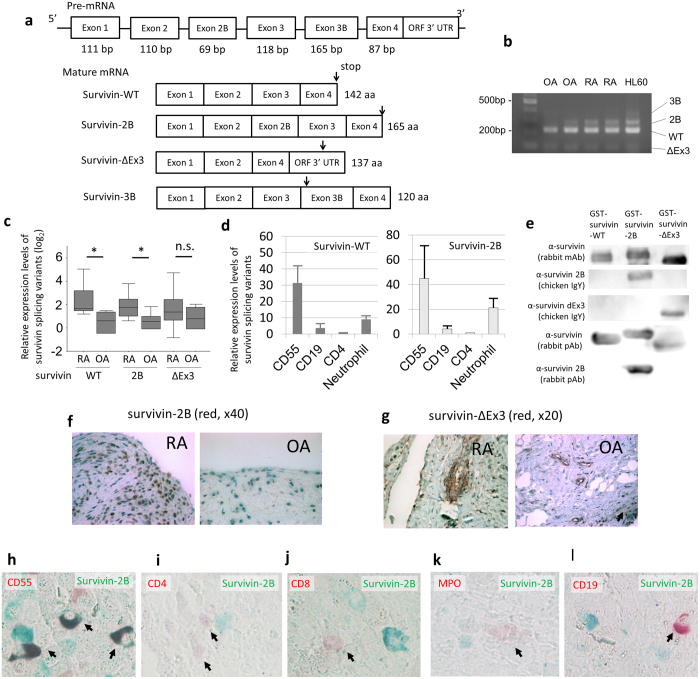
The expression of survivin splice variants in RA and OA synovial tissues.(**a**) The survivin pre-mRNA generates some mature mRNA transcripts (splice variants) which result from alternative splice. Arrows indicate the stop codon. (**b**) RT-PCR of whole synovial tissues showed the expression of survivin splice variants (n = 9 different RA patients, n = 6 different OA patients and the HL-60 cell line). The forward primer was located in Exon 2 and the reverse primer was located in Exon 4. Forty cycles of PCR were performed. The data shown are representative of the samples and cropped image was used. The gels have been run under the same experimental conditions. (**c**,**d**) The relative expression levels determined by real-time PCR with the TaqMan method. The relative expression levels were corrected based on the expression level of HPRT. (**c**) Whole synovial tissues (n = 9 from different RA patients, n = 6 from different OA patients) (RA, 68.4 ± 7.2 years old; OA, 74.1 ± 7.9 years old; t-test, p = 0.183). The Dunnett method, ^∗^p < 0.05 (WT; p < 0.01, 2B; p = 0.018 and ΔEx3; p > 0.05, respectively). (**d**) Separated cells from the synovium from different RA patients (n = 3). The CD55^+^ cells indicate RA-FLS (CD3^–^CD14^–^CD16^–^CD19^–^CD20^–^CD56^–^CD45^–^CD55^+^), the CD19^+^ cells indicate B cells (CD45^+^CD19^+^), and the CD4+ cells indicate T cells (CD45+CD4+). The results are the Means ± SEM. (**e**) The results of the Western blot analysis to confirm whether anti-survivin splice variant antibodies react with the three types of GST-fusion survivin proteins. The anti-survivin-2B (chicken and rabbit polyclonal antibodies) and anti-survivin-ΔEx3 (chicken polyclonal antibodies) antibodies recognized the GST fusion human survivin variants corresponding to each antibody. The anti-survivin antibodies (rabbit monoclonal and polyclonal antibodies) reacted against all three GST-fusion splice variants. The cropped blots were used in the figure. (**f**–**g**) Immunohistochemistrical (IHC) staining of survivin-2B and -ΔEx3 in RA and OA synovial tissues (red, x40, x20). The primary antibodies against survivin-2B and -ΔEx3 were used. (**h**–**l**) The dual IHC staining of lineage markers (red) and survivin-2B (green) in RA synovial tissues (x40). Arrows indicate lineage marker-positive cells. (**h**) CD55 and survivin-2B. (**i**) CD4 and survivin-2B. (**j**) CD8 and survivin-2B. (**k**) MPO and survivin-2B. (l) CD19 and survivin-2B.

To confirm the gene expression of survivin in whole synovial tissues, we performed reverse transcriptase-polymerase chain reaction (RT-PCR). As shown in Fig. 2b, several bands were detected, and subsequent DNA sequencing revealed the existence of three survivin variants (survivin-WT, -2B and -ΔEx3) in the synovial tissues (data not shown). The *BIRC5* gene can generate at least four splice variants, including survivin-WT, survivin-2B, survivin-ΔEx3 and survivin-3B, which result from alternative splicing^21^,^22^,^23^ (Fig. 2a). Survivin-WT, survivin-2B and survivin-ΔEx3 were all expressed in the whole synovial tissues in these patients (Fig. 2b). Survivin-3B was not detected by RT-PCR, although it was observed in the positive control (HL60 cell line).

Next, we performed a quantitative real-time PCR analysis of the three detected survivin variants (-WT, -2B and -ΔEx3) for whole synovial tissue. The expression levels of survivin-WT and -2B in RA synovial tissues were significantly higher than those in OA tissues (Fig. 2c and Supplemental Table 2). On the other hand, there was no significant difference in the survivin-ΔEx3 expression levels between RA and OA synovial tissues. Moreover, we sorted the synovial tissue specimens obtained from RA patients and performed real-time PCR. The expression levels of survivin-WT and survivin-2B in RA-FLS (CD3^−^CD14^−^CD16^−^CD19^−^CD20^−^CD56^−^CD45^−^CD55^+^ cells) were higher in other cells (Fig. 2d and Supplemental Figure 1). The expression levels of survivin-WT and survivin-2B in most of the infiltrating CD4^+^ T lymphocytes (CD45^+^CD4^+^) and CD19^+^ B lymphocytes (CD45^+^CD19^+^) were low; however some of the infiltrating neutrophils expressed higher levels than the lymphocytes.

To identify the protein expression levels of survivin splice variants in RA and OA synovial tissues, we performed IHC analyses using antibodies against the splice variants. After we proved the antibody specificity for the survivin splice variants by a Western blot analysis (Fig. 2e), serial sections of RA and OA synovial specimens were stained for IHC analyses using these antibodies against the splice variants. As shown in Fig. 2f, survivin-2B was found to be expressed in both RA and OA synoviocytes, and the expression levels of survivin-2B in RA were much higher than those in OA. Survivin-ΔEx3 was expressed mainly in the vascular endothelial cells of both RA and OA synovial tissue samples (Fig. 2g). To confirm the survivin-2B expressing cell lineage in RA synovial tissues, we performed dual staining via IHC (Fig. 2h–l). As a result, the expression of CD55 and survivin-2B coincided; therefore, survivin-2B was expressed in FLS. Conversely, most of the infiltrating neutrophils, CD19^+^ B lymphocytes, CD4^+^ and CD8^+^ T lymphocytes did not exhibit positive survivin-2B staining. Therefore, survivin-2B appears to be mainly produced from RA-FLS.

### Survivin expression and regulation in cultured RA fibroblast-like synoviocytes (RA-FLS)

**Figure 3:**
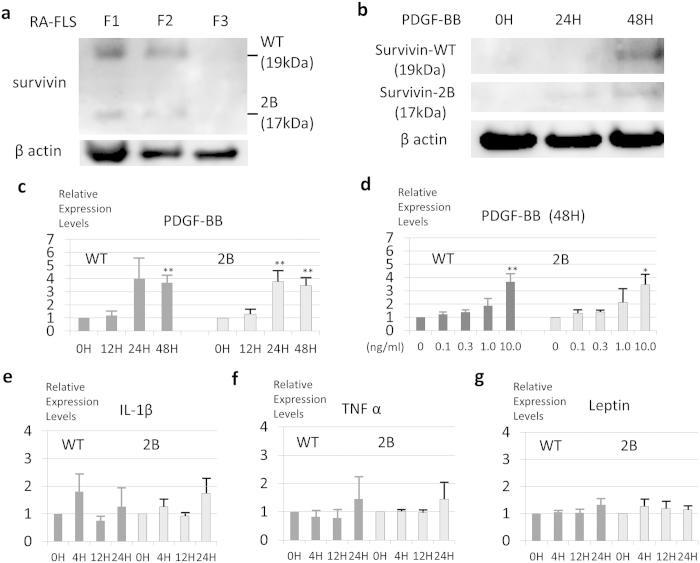
The expression levels of survivin (-WT and -2B) in RA-FLS are upregulated by PDGF, but not by IL-1β, TNF-α or leptin.(**a**) Cultured primary synoviocytes from RA patients were analyzed for survivin-WT and -2B expressions using a Western blot analysis. The survivin expression levels in the adherent cultured primary synoviocytes decreased with the cells passages. The cropped blots were used in the figure. (**b**) PDGF increased the survivin-WT and -2B expression levels, as indicated by a Western blot analysis. The cropped blots were used in the figure. (**c**–**g**) RA-FLS (n = 3 from different RA individuals) were cultured in the presence of (**c**) PDGF (10 ng/ml), (**e**) IL-1β (1 ng/ml), (**f**) TNF-α (20 ng/ml) or (**g**) leptin (1000 nM) for 24 to 48 hours and (**d**) PDGF (from 0.1 to 10.0 ng/ml) for 48 hours, after serum starvation (0.1%FBS/DMEM) for 24 hours. The time- and dose-dependent relative expression levels of survivin-WT and -2B were analyzed by real-time PCR. The relative expression levels were corrected based on the level of HPRT. Adherent FLS were used for experiments (without (**a**)) at passages 3 through 6. The results are the Means ± SEM (standard error of the mean). The Dunnett method, ^∗∗^p < 0.01, ^∗^p < 0.05.

Next, the expression of survivin in cultured RA fibroblast-like synoviocytes (RA-FLS) was investigated. As shown in Fig. 3a, the expression levels of survivin-WT and -2B in the RA-FLS were gradually decreased with serial passages, as determined by a Western blot analysis. Therefore, it is conceivable that the survivin-WT and -2B expression in RA-FLS might be unstable and maintained by some soluble factors. To determine the factor(s) responsible for survivin expression in RA-FLS, we examined the effects of various candidate soluble factors, including IL-1β, TNF-α, leptin and PDGF.

As shown in Fig. 3c–d, the expression levels of survivin-WT and -2B in the RA-FLS were upregulated by PDGF in both time- and dose-dependent manners. PDGF-induced survivin expression was also observed at the protein level, as determined by a Western blot analysis (Fig. 3b). On the contrary, other soluble factors (such as TNF-α, IL-1β and leptin) did not affect the expression of survivin in the RA-FLS (Fig. 3e–g).

### Survivin-2B induces RA-FLS proliferation

**Figure 4:**
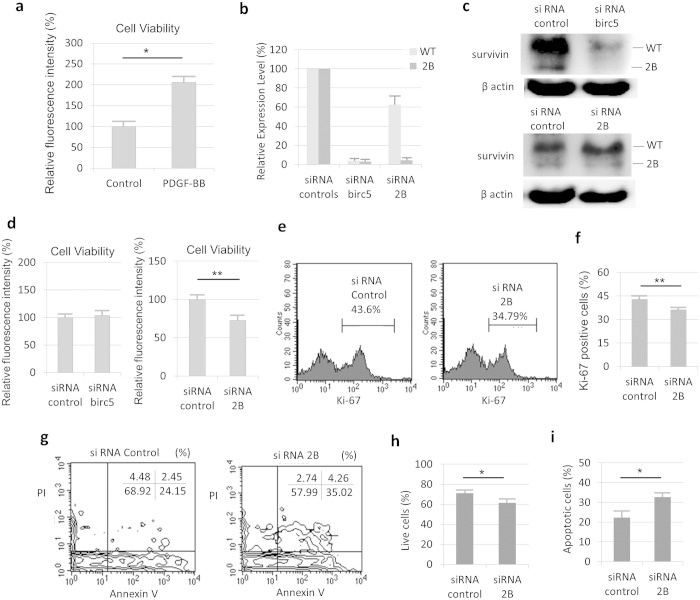
Functional analyses using siRNA against survivin-2B in PDGF treated RA-FLS. (**a**) The calcein assay for cell proliferation was performed. PDGF increased the number of RA-FLS at 36hours (n = 4, p < 0.05). (**b**–**i**) RA-FLS treated with PDGF (10 ng/ml) and transfected with siRNA. (**b**,**c**) The siRNA birc5 (all survivin isoform silencer) decreased the expression levels of both survivin-WT and -2B. The siRNA 2B decreased the expression level of survivin-2B and had little influence on the survivin-WT expression. (**b**) The results of real-time PCR for siRNA-induced survivin silencing (n = 3). (**c**) The results of Western blot analyses for siRNA-induced survivin silencing with siRNA birc5 (upper) or siRNA 2B (lower). The cropped blots were used in the figure. (**d**) The viability of RA-FLS transfected with siRNA birc5 (left, n = 5) or siRNA 2B (right, n = 6) at 48 hours as determined using the calcein assay. (**e**,**f**) Measurement of Ki-67-positive RA-FLS transfected with siRNA at 48hours (n = 3). The positive rate was calculated as the number of Ki-67-positive cells among the total RA-FLS. (**e**) FACS plots for siRNA control (left) and siRNA 2B (right). (**g**–**i**) The annexin V and PI analysis for RA-FLS transfected with siRNA at 48hours (n = 4). H_2_O_2_ stimulation (0.1 mM) was performed for 3 hours before the analysis. (**g**) FACS plots for siRNA control (left) and siRNA 2B (right). (**h**) The percentage of live cells was calculated as the number of annexin V- and PI-double negative cells among the total RA-FLS. (**i**) The percentage of apoptotic cells was calculated as the number of annexin V-positive and PI-negative cells among the total RA-FLS. The results are the Means ± SEM. t-test ^∗^p < 0.05, ^∗∗^ p < 0.01.

PDGF has been reported to enhance the proliferation of RA-FLS^24^,^25^. To confirm the effects of PDGF, a cell viability assay was performed using RA-FLS. After RA-FLS were treated with PDGF, the cell number was higher than that of the cells in control medium (Fig. 4a).

Subsequently, to clarify the involvement of survivin in the PDGF-induced RA-FLS proliferation, siRNA transfection experiments were performed. RA-FLS were transfected with siRNA against survivin (siRNA birc5 (all survivin isoform silencer)) or survivin-2B (siRNA 2B (specific silencer)) under PDGF treatment (Fig. 4b–c). The RA-FLS were treated with both PDGF and each of the siRNAs, and were evaluated using a cell viability assay. As shown in Fig. 4d, the number of siRNA 2B-treated FLS was lower than siRNA control. On the other hand, siRNA birc5-treated FLS did not affect the cell number. Therefore, survivin-2B specific silencing led to suppression of the RA-FLS cell viability.

Next, we investigated whether survivin-2B silencing might affect the cell cycle and hydrogen peroxide-induced apoptosis in RA-FLS treated with both PDGF and siRNA 2B. The Ki-67-positive RA-FLS, which appear during the active phase of cell cycle, were lower in the cells transfected with siRNA 2B than in those transfected with siRNA control (Fig. 4e–f). In addition, the number of annexin V-positive and propidium iodide (PI)-negative RA-FLS, which indicated cells in the apoptotic stage, was higher, and the number of live cells (annexin V and PI-double negative) was lower in the cells transfected with siRNA 2B than those transfected with siRNA control (Fig. 4g–i). Therefore, survivin-2B silencing in RA-FLS led to suppression of the cell cycle and promoted apoptosis.

**Figure 5:**
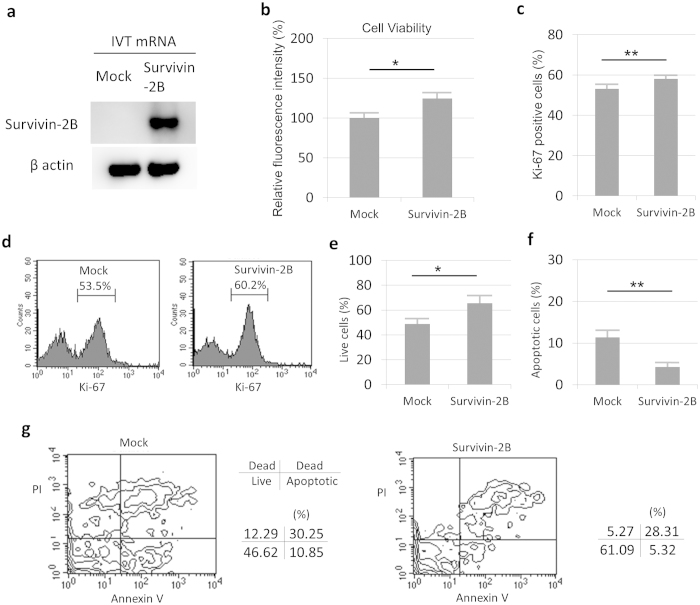
Functional analyses of survivin-2B-overexpressing RA-FLS.The IVT mRNA (mock and survivin-2B) transfection was used for overexpression experiments for RA-FLS. (**a**) A Western blot analysis showed that survivin-2B mRNA expressed survivin-2B protein. The cropped blots were used in the figure. (**b**) The viability of RA-FLS transfected with mRNA (n = 6) at 36hours as determined using the calcein assay. (**c**,**d**) Measurement of the Ki-67-positive RA-FLS transfected with mRNA at 48hours (n = 4). The positive rate was calculated as the number of Ki-67-positive cells among the total RA-FLS. (**d**) FACS plot for mock mRNA (left) and survivin-2B mRNA (right). (**e**–**g**) The annexin V and PI analysis for RA-FLS transfected with mRNA at 24hours (n = 4). H_2_O_2_ stimulation (0.1 mM) was performed for 3hours before the analysis. (**e**) The percentage of live cells was calculated as the number of annexin V- and PI-double negative cells among the total RA-FLS. (**f**) The percentage of apoptotic cells was calculated as the number of Annexin V-positive and PI-negative cells among the total RA-FLS. (**g**) FACS plot for mock mRNA (left) and survivin-2B mRNA (right). The results are the Means ± SEM. t-test ^∗^p < 0.05, ^∗∗^ p < 0.01.

Moreover, we performed overexpression experiments in RA-FLS with IVT mRNA transfection and also confirmed the contribution of survivin-2B in the cell cycle and hydrogen peroxide-induced apoptosis (Fig. 5a). As shown in Fig. 5b, the number of survivin-2B-overexpressing FLS was higher than in control mRNA transfected cells, which were evaluated using a cell viability assay. Furthermore, the number of Ki-67-positive cells was higher in the survivin-2B-overexpressing cells than in the control cells (Fig. 5c–d), and the number of apoptotic cells was lower in the survivin-2B-overexpressing FLS than control cells (Fig. 5e–g). These data suggest that survivin-2B contributes to RA-FLS proliferation through cell cycle activation and apoptosis inhibition.

### Survivin-2B is also detectable in the sera from RA patients

**Figure 6:**
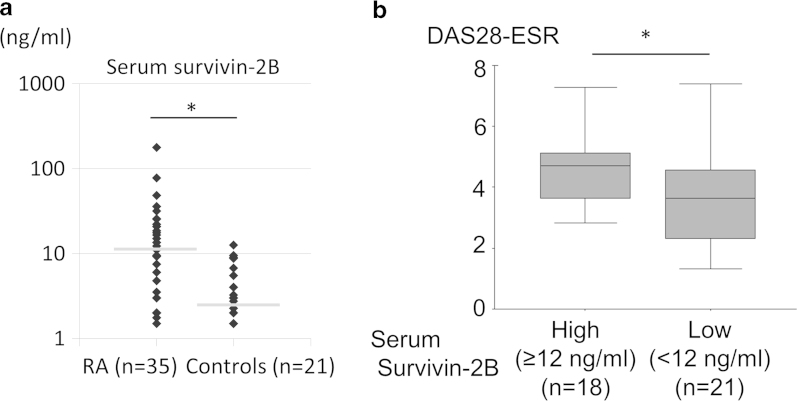
The serum expression levels of survivin-2B in RA patients determined using an ELISA. (**a**) We collected serum samples from 35 RA patients and 21 age- and sex- matched controls (healthy controls and OA patients), and measured the serum survivin-2B levels with an ELISA (mean RA, 18.7 ng/ml vs. controls, 4.7 ng/ml; p = 0.049) (RA, 64.7 ± 11.4 years old; controls, 59.6 ± 18.0 years old; p = 0.250). The gray lines indicate the median of survivin-2B level in each group. (**b**) We compared the DAS28-ESR between the survivin-2B high RA group (n = 18) and the survivin-2B low RA group (n = 21) (mean RA, 4.64 vs. controls, 3.64; p = 0.037). t-test, ^∗^p < 0.05.

To clarify the clinical importance of survivin-2B in RA, we developed a survivin-2B-specific ELISA, and measured the serum concentrations of survivin-2B in RA and control subjects. As shown in Fig. 6a and Supplemental Table 3, serum survivin-2B concentrations in RA patients (n = 35) were significantly higher than those in controls (n = 21) (p = 0.049), which exhibited a similar pattern to the serum survivin concentrations, as indicated by the ELISA (Fig. 1a).

When the mean + 2SD (standard deviation) was defined as the cut-off value for survivin-2B, the calculated cut-off level was 12** **ng/ml. We divided the RA patients into two groups based on this cut-off level: survivin-2B high (≥12** **ng/ml) (n = 18) and survivin-2B low (<12** **ng/ml) (n = 21). As a result, the survivin-2B high RA patients had higher disease activity score (DAS28-ESR) than the patients with low levels of survivin-2B (average 4.64 vs 3.64) (p = 0.037) (Fig. 6b and Table 1). These data suggest that the serum survivin-2B concentration might reflect the disease activity of RA.

## Discussion

In the present study, we demonstrated the expression and function of survivin-2B in the human RA synovial tissue. This report is the first publication showing the relationship between survivin-2B and RA.

The proto-oncogene survivin has attracted increasing attention as a therapeutic target for cancer since 2002, when its gene transcription was reported to be repressed by wild-type p53^26^,^27^,^28^. It has been reported that some normal adult tissues also expresses survivin, including the synovial tissue, primitive hematopoietic cells, T lymphocytes and polymorphonuclear neutrophils^16^,^19^,^20^. The present research started with an investigation of the survivin expression levels in blood and synovial tissues from RA patients and control subjects. As a result, we found that both serum and synovial survivin expression levels in RA patients were higher than those in controls. We believe that the origin of the survivin detected in the RA patient serum may be mainly from the synovial tissues, in particular FLS.

The *BIRC5* gene generates at least four splice variants, including survivin-WT, survivin-2B, survivin-ΔEx3 and survivin-3B, which result from alternative splicing^21^,^22^. Additionally, the expression of other splice variants survivin, including -2α, -2B+32, -2B+32/3B, -ΔEx2 and so on have been reported in human tissues^23^,^29^,^30^. Human and non-human primates have splice variants which have additional sequences (for example, exon 2B or exon 3B)^29^, while, murine survivin variants are mainly deletion splicing isoforms (for example, survivin140 (WT), survivin121 and survivin40), and survivin-2B expression has never been reported in mice^31^.

Survivin-2B has already been reported in many types of human malignant tumors: soft tissue sarcoma, gastric carcinoma, brain tumor, hepatocellular carcinoma, non-small-cell lung cancer (NSCLC) and acute myeloid leukemia (AML)^30^,^32^,^33^,^34^,^35^,^36^,^37^. In the present study, we were able to detect survivin-2B expression in RA patients’ sera and synovial tissues. When we compared RA and OA specimens using IHC, the survivin-2B expression levels of synoviocytes in RA patients were much higher than those in OA patients. The expression levels of survivin-2B in RA cases were extremely high, while the expression levels in OA patients were low. Accordingly, we suppose that survivin-2B might be an attractive tissue marker of RA synovitis. Additionally, the main origin of survivin-2B expression was FLS, as demonstrated with real-time PCR and dual IHC staining. Simultaneously, infiltrating neutrophils may also be a candidate as the source for survivin-2B expression. However, the dual IHC staining revealed that the protein expression levels in neutrophils were not high. We therefore speculate that the main source of survivin-2B is FLS.

In our investigation, we also performed an ELISA against survivin (common) and survivin-2B. The levels of both of these proteins in the RA patients’ sera were higher than those in control subjects, and exhibited a similar pattern. Therefore, the expression of survivin-2B may contribute to the increase in the survivin concentration in RA patients’ blood. Additionally, the survivin-2B high RA patients had higher disease activity than did the survivin-2B low RA patients. We suspect that survivin-2B-expressing proliferative synoviocytes lead to higher swollen and tender joint scores, which may result in a higher DAS28-ESR. We believe that serum survivin-2B has the potential to be used as a biomarker for RA.

It has been unclear which factors can increase the survivin-2B expression level. Leptin and several growth factors have already been reported as factors that can induce survivin-WT expression^38^,^39^,^40^. As shown by our present results, the expression of survivin-2B in the RA-FLS was upregulated by PDGF, and was not affected by IL-1β, TNFα or leptin. PDGF is produced from synovial cells and platelets, and has an important role in RA-FLS proliferation^24^,^25^. Therefore, the fact that PDGF can induce survivin, a factor associated with RA joint destruction, might imply a significant clue to the pathogensis of RA.

Survivin not only inhibits apoptosis as an endogenous caspase inhibitor, but also regulates cell division in the G2/M phase, which makes it unique among the IAPs^41^. Several survivin variants can form homodimers and heterodimers comprising two different splice variants, which affect the functions of survivin^42^. The function of survivin-2B has been controversial. The expression levels of survivin-2B in solid tumors were often negatively correlated with the clinical stages of malignant tumors and the recurrence rates^33^,^34^,^36^. Therefore, these authors postulated that survivin-2B might diminish survivin-WT function in a dominant negative manner and could have pro- functions in solid tumors. On the other hand, the high expression of survivin-2B in AML correlated with a decreased overall survival, so survivin-2B might have an anti-apoptotic function^37^.

The functions of survivin and its splice variants in RA-FLS have never been reported. Therefore, we herein performed both siRNA and IVT mRNA transfection experiments in RA-FLS to evaluate its function. As a result, survivin-2B silencing decreased the cell viability of RA-FLS treated with PDGF via cell cycle suppression and by promoting apoptosis while survivin-2B-overexpression increased the cell viability of RA-FLS via cell cycle promotion and by inhibition of apoptosis. Therefore, survivin-2B contributes to the proliferation of RA-FLS through cell cycle activation and by conferring resistance to apoptosis. We suppose that survivin-2B is a key molecule involved in rheumatoid synovial hyperplasia, and that it has the potential to be a novel therapeutic target for RA.

Interestingly, siRNA birc5 (all survivin isoform silencer), which we expected to be an inhibitor of all birc5 mRNA, could not suppress the PDGF-induced RA-FLS proliferation. We suspect that a dominant negative (DN) isoform of some types of survivin splice variant may have existed in RA-FLS. Candidate DN isoforms could include survivin-WT or unknown variants. The siRNA against total birc5 could silence both survivin-2B and the DN isoform, therefore survivin silencing may not lead to the inhibition of RA-FLS viability. Accordingly, when we select survivin as a therapeutic target for rheumatoid synovial hyperplasia, survivin-2B-specific silencing might be more appropriate than all survivin isoform silencing. For example, survivin-2B-specific siRNA treatment using nanoliposomes has already been reported in an orthotopic murine model of ovarian cancer^43^.

It is also necessary to pay attention to the relationship between tumor immunity and survivin-2B. In some HLA-A*2402-positive patients suffering from oral cancers, survivin-2B 80-88-specific cytotoxic T-lymphocytes (CTL) were detected^44^. The survivin-2B 80-88 peptide (amino acid sequence AYACNTSTL) can bind to the HLA-A24 molecule and was capable of inducing CTLs which could kill HLA-A24-positive cancer cells^45^. HLA-A*2402 is one of the most frequently detected alleles in the HLA class I genes. Therefore, the impact of survivin-2B on HLA-A24-positive RA-FLS may activate synovial CTLs and promote synovial inflammation. Similarly, survivin-2B silencing might be able to regulate synovial CTL activity.

In conclusion, the expression of survivin-2B was detected in blood and synovial tissues from RA patients. Survivin-2B was mainly expressed in RA-FLS. Survivin-2B was up-regulated by PDGF, and contributed to the proliferation of RA-FLS. Survivin-2B is therefore considered to be potentially useful as a biomarker, and it may thus represent a novel therapeutic target for RA.

## Methods

### Preparation of human blood, synovial tissue and FLS

This study was approved by the clinical ethics committees of Dohgo Spa Hospital and the Department of Pathology, Ehime University Proteo-Science Centre and Graduate School of Medicine, and was conducted at these institutions. The methods were carried out in accordance with the approved guidelines. We collected synovial tissues and blood samples from RA patients who fulfilled the classification criteria of the ACR 1987^46^, and also collected samples from normal subjects and OA patients as controls at Dohgo Spa Hospital. Synovial tissues were obtained from sixteen RA patients and ten OA patients who were undergoing open synovectomy or total joint replacement after the subjects provided their informed consent and signed a written consent form. Blood samples were obtained from 61 RA patients and 37 controls (including OA patients), who also had provided their informed consent and signed a written consent form. These samples were stored at −80** **°C until they were analyzed. The RA disease activity was determined based on the DAS28-ESR according to the formula on the DAS (Disease Activity Score) website^47^. For synovial tissue sorting and RA-FLS preparation, synovial tissues from five different RA patients were minced and incubated with 1** **mg/ml collagenase/dispase (Roche) in PBS (pH7.2, Gibco) for 1 hour at 37°C, then were filtered and washed. For RA-FLS preparation, synovial cells were diluted and cultured. During culturing, the supernatant replaced frequently to remove nonadherent cells. Adherent FLS were split at 1:3 when 80% confluent and passaged. FLS from RA patients were cultured in DMEM (Gibco) supplemented with 10% fetal bovine serum (FBS) (Gibco) and penicillin/streptomycin (Gibco). FLS were used for experiments (except Fig. 3a) at passages 3 through 6 and were starved in DMEM with 0.1% FBS for 24 hours before the addition of human recombinant cytokines.

### Synthesis of survivin and the splice variant proteins using a cell-free system

Artificial custom genes encoding human survivin, including the wild type (WT) protein and -2B and -ΔEx3 variants, were synthesized by Integrated DNA Technologies Inc., USA. The sequences of these genes corresponded to GenBank accession Nos. NM 001168.2 (WT), NM 001012271.1 (2B) and NM 001012270.1 (ΔEx3), respectively. The proteins for the survivin variants were prepared using a cell-free protein synthesis system with wheat germ ribosomal RNA^48^,^49^. The custom synthesized genes were inserted into pEU-E01G expression vectors (CellFree Sciences, Matsuyama, Japan) containing a glutathione-S-transferase (GST) region and a SP6 promoter. The GST fusion survivin variant proteins were automatically synthesized using a Robotic Protein Synthesizer Protemist® DT (Cell Free Sciences), as described previously^50^.

### Recombinant proteins and antibodies

Recombinant human interleukin-1β (IL-1β) (R&D systems), tumor necrosis factor-α (TNFα) (R&D systems), leptin (Sigma Aldrich) and dimeric platelet-derived growth factor composed of two B units (PDGF-BB) (Sigma Aldrich) were purchased for the experiments. For the immunohistochemistry (IHC) studies, Western blot analysis and enzyme-linked immunosorbent assay (ELISA), we purchased the following antibodies: anti-survivin rabbit polyclonal antibody (R&D systems), anti-survivin rabbit monoclonal antibody (Cell Signaling Technology, clone. 71G4B7), anti-suvivin-2B rabbit polyclonal antibody (Abcam), anti-survivin-2B and anti-survivin-ΔEx3 chicken IgY antibodies (CH3 BioSystems), anti-β-actin antibody (Sigma-Aldrich, clone. AC-15), anti-CD55 mouse monoclonal antibody (Abcam, clone. 67), anti-human CD4 mouse monoclonal antibody (eBioscience, clone. CRRY77), anti-CD8a mouse monoclonal antibody (eBioscience, clone. C8/144B), anti-CD19 mouse monoclonal antibody (eBioscience, clone. LC1), anti-MPO mouse monoclonal antibody (Nichirei, clone. 59A5), horseradish peroxidase (HRP)-conjugated goat anti-rabbit IgG antibody (Jackson Immuno Research) and HRP-conjugated donkey anti-chicken IgY antibody (Jackson Immuno Research). The HRP-conjugated polymer anti-rabbit IgG antibody (Envision plus reagent, Dako) and the alkaline phosphatase (AP)-conjugated polymer anti-mouse IgG antibody (Simplestain AP, Nichirei) were used as secondary antibody of IHC. For fluorescence-activated cell sorting (FACS), antibodies conjugated with FITC, PE, PerCP or allophycocyanin (APC) were used. Anti-human CD8 (clone. T8) and human CD14 (Mo2) antibodies were purchased from Beckman Coulter. Anti-human CD45 (2D1) and lineage cocktail 1 (lin1) (CD3, CD14, CD16, CD19, CD20, CD56) were obtained from BD Biosciences. Anti-human CD4 (OKT4), anti-human CD19 (SJ25C1), anti-human CD55 (143-30), anti-human Ki-67 (Ki-67) antibodies, Annexin V and PI were obtained from eBioscience.

### Flow cytometry

The flow cytometric analysis was performed using a FACS Aria II and Calibur instrument (BD biosciences). For cell sorting, cells were washed and stained. For Ki-67 staining of RA-FLS, FOXP3 Staining Buffer Set (eBioscience) was used. Cells were washed, dissociated with trypsin, permeabilized and stained. For the Annexin V staining of RA-FLS, Annexin V Apoptosis Detection kit (eBioscience) was used. Cells were washed, dissociated with trypsin and stained.

### Enzyme-linked immunosorbent assay (ELISA)

The serum survivin levels were measured with a Quantikine ELISA kit for human survivin (R&D systems) according to the manufacturer’s protocol. The survivin-2B levels were determined by a sandwich ELISA. First, 96-well polystyrene microplates coated with a mouse monoclonal antibody against human survivin, which were obtained from R&D systems, were prepared. The plates were blocked with blocking solution, then patients’ serum samples were added into the wells. Following several washes with TBST, an anti-survivin 2B antibody (rabbit polyclonal), then a goat anti-rabbit IgG-HRP and the corresponding substrate were used to assess the expression of the variant. The double-wavelength readings at 450 and 570** **nm were used, and the differences in absorbance were calculated. The standard absorbance value was compared with those of recombinant survivin-2B, as described above.

### Immunohistochemistry (IHC)

Sections obtained from formalin-fixed paraffin-embedded tissues were used for IHC. Following deparaffinization and antigen retrieval in Tris-EDTA buffer (pH 9.0), the specimens were incubated with primary antibodies, followed by incubation with the HRP- or AP-conjugated secondary antibodies, then the samples were visualized using the AEC plus substrate-chromogen (Dako), Fuchsin+ Substrate-Chromogen (Dako) and Histogreen Substrate kit for peroxidase (Abcys). The slides were counterstained with hematoxylin solution.

### RNA extraction and quantitation of the survivin expression levels in synovial tissue specimens

The expression levels of survivin and its splice variants were assessed in sorted cells, RA-FLS, RA and OA synovial tissues. The HL-60 cell line was used as a positive control for the survivin variants. The total RNA from each cell or the whole synovial tissues was extracted and purified using the Trizol reagent (Life Technologies) followed by cDNA synthesis using a PrimeScript RT Reagent Kit with gDNA Eraser (Takara-Bio). The primer pair used to amplify human survivin (-WT, -ΔEx3, -2B and -3B) consisted of a forward primer, 5′-CCCAGTGTTTCTTCTGCTTCAA-3′ and a reverse primer, 5′- TCCGCAGTTTCCTCAAATTCTT-3′. Quantitative real-time PCR using Taqman probes (FAM/TAMRA) was executed in triplicate, and was carried out on ABI 7500 Real-time PCR system (Applied Biosystems). The expression of human hypoxanthine phosphoribosyltransferase (HPRT) was evaluated as a housekeeping gene to normalize the ΔCt values. The relative expression levels of the target genes were obtained using the differences based on in the comparative threshold (ΔΔCt) method. For analyses of sorted specimens, the Ct values of each gene from CD45+CD4+ cells were applied as the standard Ct values. The upstream and downstream primer sequences used for survivin-WT and the fluorogenic probe that comprised a sequence located between the PCR primers were 5′-CCCCATAGAGGAACATAAAAAGC-3′, 5′-GGTTTCCTTTGCAATTTTGTTCT-3′ and 5′-ATTCGTCCGGTTGCGCTTT-3′, respectively. The upstream and downstream primer sequences for survivin-2B and the fluorogenic probe were 5′- ATGACGACCCCATTGGGCC-3′, 5′- TTTTTATGTTCCTCTCTCGTGATCCG-3′ and 5′- CGCCTGTAATACCAGCACTTTGGGAGGCC-3′, respectively. The upstream and downstream primer sequences for survivin-ΔEx3 and the fluorogenic probe were 5′-GACGACCCCATGCAAAGGAAAC-3′, 5′-CTCAATCCATGGCAGCCAGCT-3′ and 5′- GAGGAAACTGCGGAGAAAGTGCGCCGT-3′, respectively.And the upstream and downstream primer sequences for human HPRT and the fluorogenic probes were 5′-GGCAGTATAATCCAAAGATGGTCAA-3′, 5′-GTCTGGCTTATATCCAACACTTCGT-3′ and 5′-CAAGCTTGCTGGTGAAAAGGACCCC-3′, respectively.

### Western blot analysis

Cells were plated in six-well plates and were washed with PBS before collection. Ten micrograms each of synthesized protein or of protein from cultured cells was processed using a NuPAGE 4-12% precast gel, and was transferred to a polyvinylidene difluoride membrane. The blotted membranes were incubated with the primary antibodies, followed by horseradish peroxidase (HRP)-conjugated secondary antibodies. Membranes were developed with the ECL prime reagents (GE Healthcare) and imaged on an Image Quant LAS 4000 mini (GE Healthcare).

### siRNA transfection

A total of 3 × 10^4^ or 3 × 10^3^ FLS per a well in 24 or 96-well plates were transfected with siRNA at 10-30 or 1-3** **pmol using ScreenFect A (Incella), according to the manufacturer’s protocol. The siRNA targeting birc5 (all survivin isoform silencer) (#6546) and the negative control (#6568) were purchased from Cell Signaling Technology. The siRNA targeting survivin-2B [3′-GCUUACGCCUGUAAUACCAdTdT (sense) and 5′-UGGUAUUACAGGCGUAAGCdTdT (antisense)] (Custom siRNA Synthesis) and the negative control (SIC-001) were obtained from Sigma-Aldrich.

### Generation and transfection of *in vitro*-transcribed (IVT) mRNA

The methods of generation and transfection of IVT mRNA were described previously^51^,^52^. To generate templates for IVT mRNA, we developed the pcDNA3-A(124) vector plasmid from artificial custom genes, which were synthesized by GenScript USA Inc. The *in vitro* transcription of pcDNA3-A(124)-based plasmids was carried out with the mMessage mMachine T7 transcription Kit (Ambion). After *in vitro* transcription, IVT mRNA was purified using the MEGAclear Kit (Ambion). To transfer IVT mRNA into cells, a total of 3 × 10^5^ FLS were transfected with 5ug of mRNA using a human dermal fibroblast nucleofector kit, protocol U-023 (Amaxa).

### Evaluation of cell growth

To evaluate the cell growth of RA-FLS, the cell viability was evaluated using the Cell Counting Kit-F (CCK-F, Dojindo, Japan). A total of 3 × 10^3^ FLS per well in 96-well plates were transfected with siRNA and IVT mRNA. After incubation of the cells for 36-48** **hours at 37** **°C, 100** **μl aliquots of supernatant were replaced with new medium, CCK-F was added, and the cells were incubated for 30 minutes at room temperature. After that, the calcein fluorescence intensities for cell viability determination were measured. The excitation and emission wavelengths of calcein were 485** **nm and 535** **nm, respectively.

### Statistical analysis

The significance of differences between two groups was determined by unpaired t-test. Contingency table analyses were performed using the chi-square test. The differences among real-time PCR expression levels were estimated using the Kruskal-Wallis test followed by the Dunnett method. The data processing and analyses were conducted using the Microsoft Excel software program.

## Additional Information

**How to cite this article**: Mokuda, S. *et al*. The proto-oncogene survivin splice variant 2B is induced by PDGF and leads to cell proliferation in rheumatoid arthritis fibroblast-like synoviocytes. *Sci. Rep.* **5**, 9795; doi: 10.1038/srep09795 (2015).

## Supplementary Material

Supplementary Information

## Figures and Tables

**Table 1:** The baseline characteristics of the serum survivin-2B measurements using ELISA in RA patients.

	**Survivin-2B high (≥12 ng/ml)**	**Survivin-2B low (<12 ng/ml)**	**p-value**
	**(n** = **18)**	**(n** = **21)**	
Age	68.9 ± 14.3	66.6 ± 9.7	0.553*
Sex (Female/Male)	F 17/M 1	F 19/M 2	0.643†
Duration of RA (year)	10.8 ± 9.2	12.6 ± 9.9	0.560*
Dose of glucocorticoids (prednisolone, mg/day)	3.2 ± 2.1	3.2 ± 2.9	0.973*
Current use of non-biologic DMARDs	15 (83.3%)	20 (95.2%)	0.246†,‡
Current use of biologics	7 (38.9%)	7 (33.3%)	0.718†,§
Disease activity score of RA (DAS28-ESR)	4.64 ± 1.10	3.64 ± 1.67	0.037**

Mean ± Standard Deviation (SD).

DMARDs, disease modifying anti-rheumatic drugs.

^*^t-test, * p < 0.05.

^†^chi-square test.

^‡^Non-biologic DMARDs included methotrexate, tacrolimus, salazosulfapyridine, leflunomide, D-penicillamine, bucillamine.

^§^Biologics included etanercept, infliximab, golimumab, abatacept and tocilizumab.
