# Supplementary material for: The proto-oncogene survivin splice variant 2B is induced by PDGF and leads to cell proliferation in rheumatoid arthritis fibroblast-like synoviocytes
Source: Sci Rep. 2015 May 22;5:9795. doi: 10.1038/srep09795 (PMC4441133; doi:10.1038/srep09795)
Supplement: Supplementary Information [file srep09795-s1.pdf]

Supplementary Information file

Title of the manuscript: The proto-oncogene survivin splice variant 2B is induced by PDGF and leads to cell proliferation in rheumatoid arthritis fibroblast-like synoviocyte

Full-length gels and blots
Supplemental Table 1-3
Suppleental Figure 1
are included.

Authors: Sho Mokuda*, Tatsuhiko Miyazaki, Yuki Ito, Satoshi Yamasaki, Hiroko Inoue, Yun Guo, Weng-Sheng Kong, Masamoto Kanno, Kiyoshi Takasugi, Eiji Sugiyama and Junya Masumoto.

Figure 2b

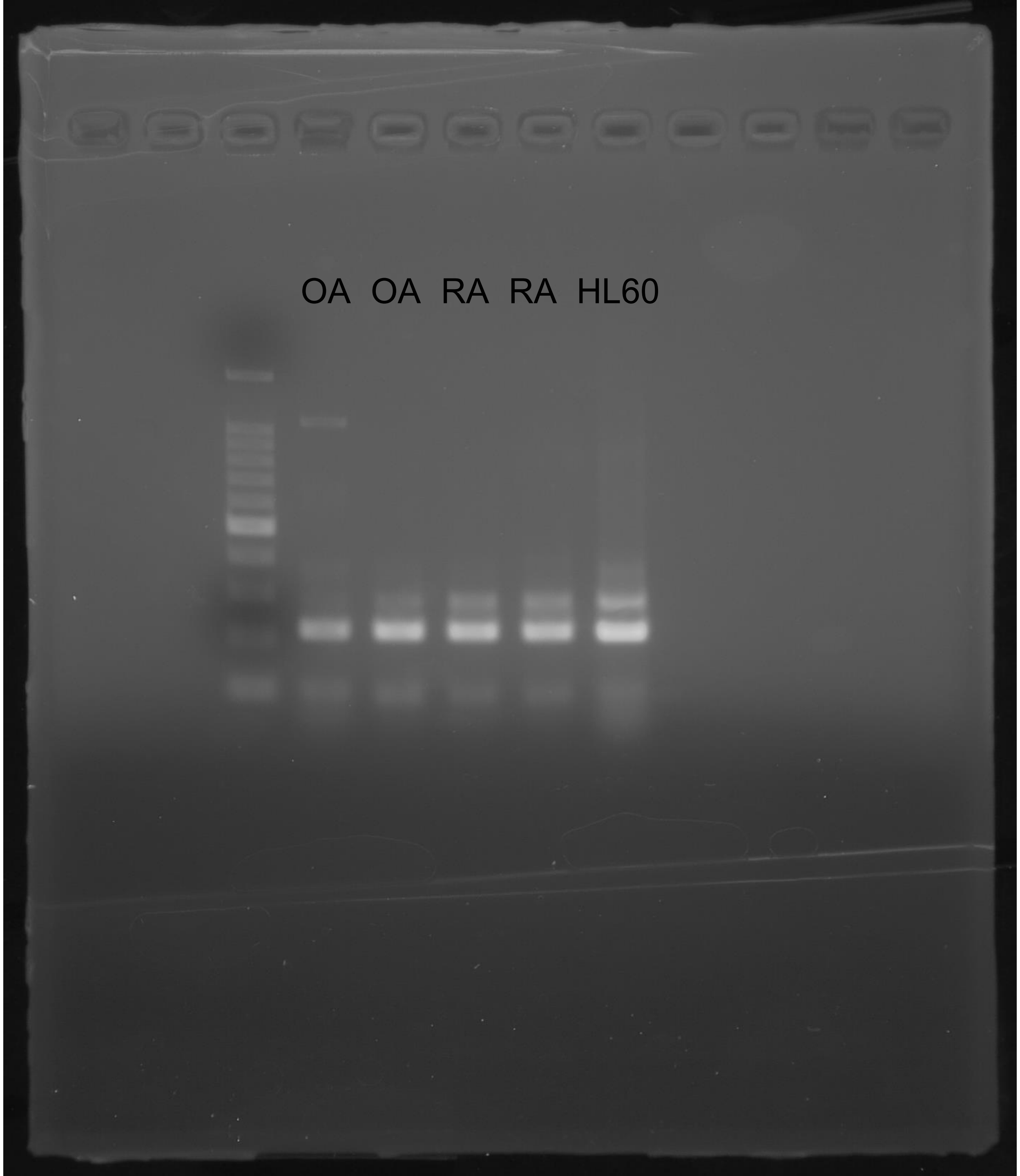

Figure 2e

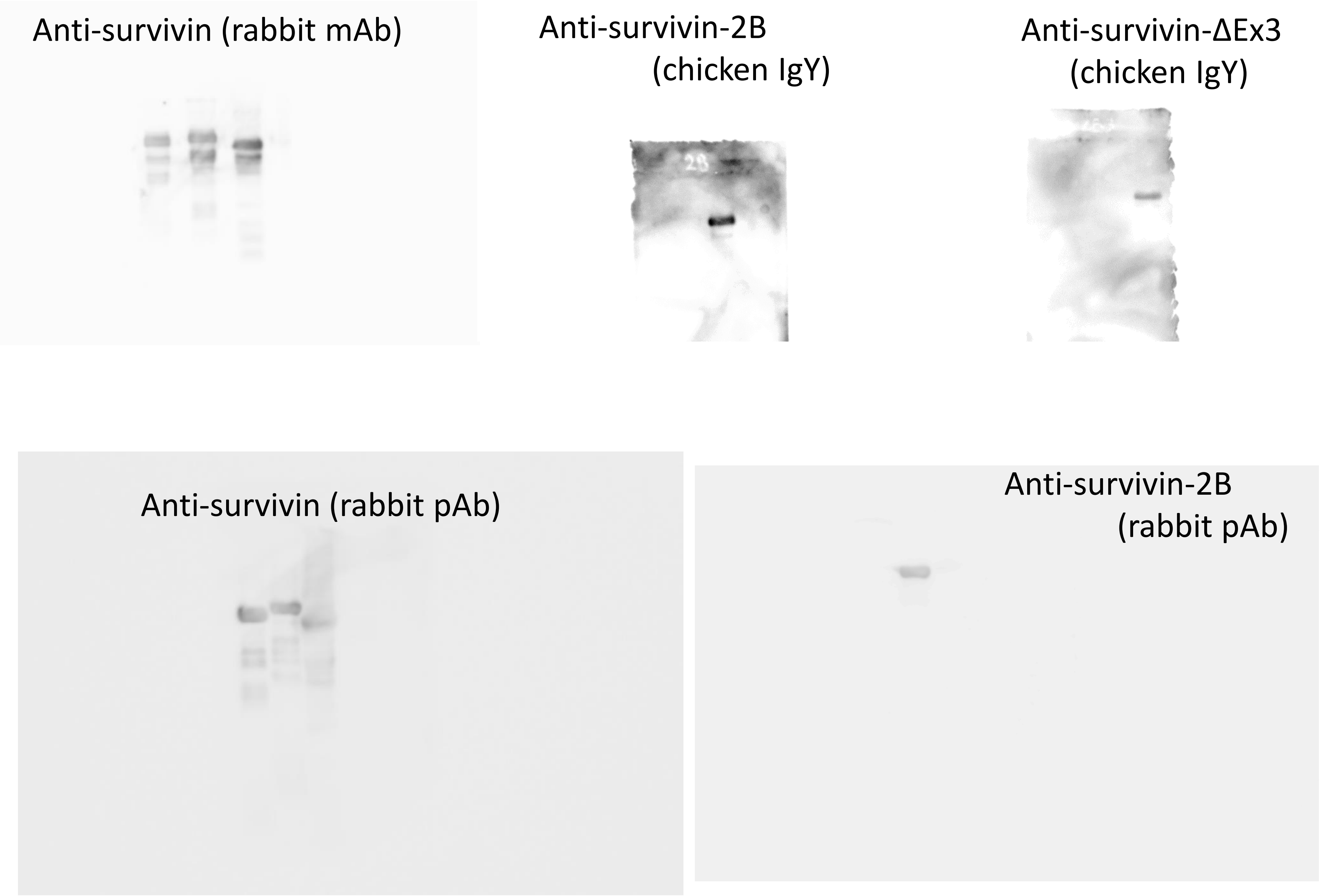
Anti-survivin (rabbit mAb)
Anti-survivin-2B
(chicken IgY)
Anti-survivin-ΔEx3
(chicken IgY)
Anti-survivin (rabbit pAb)
Anti-survivin-2B
(rabbit pAb)

Figure 3a

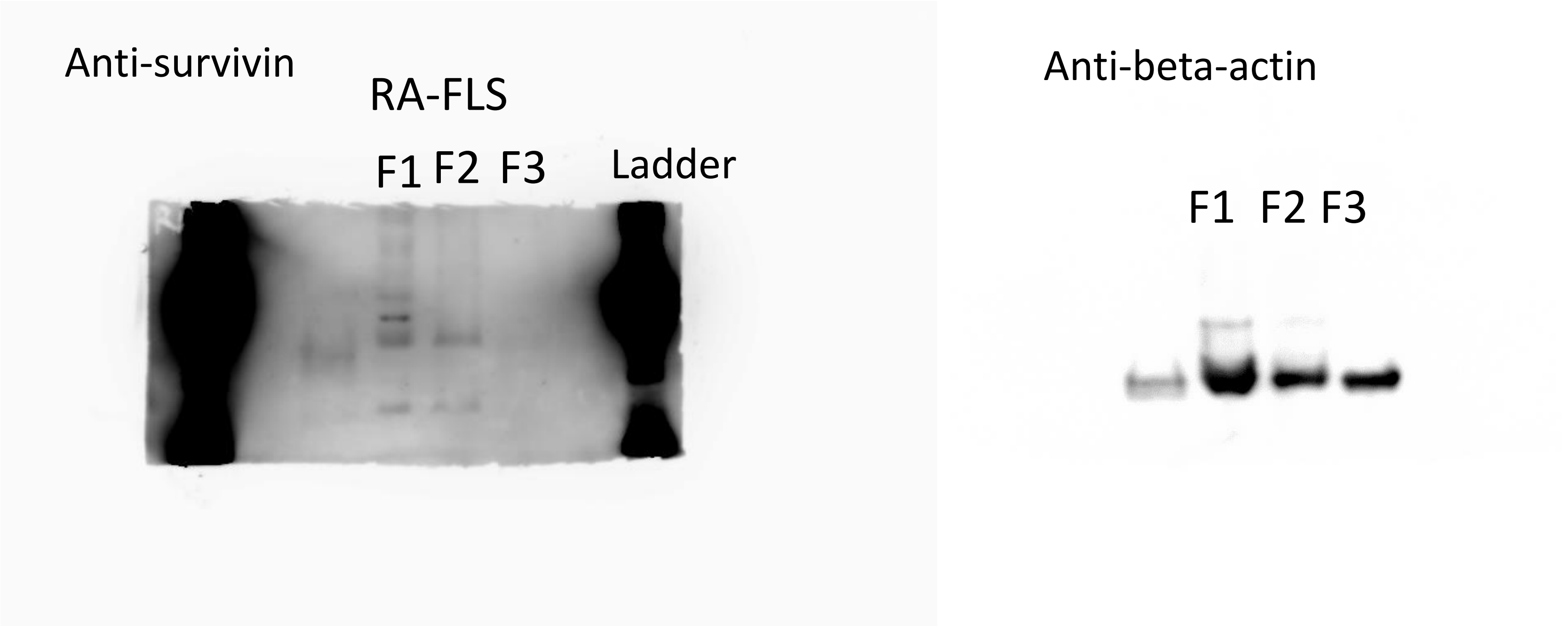

Figure 3b

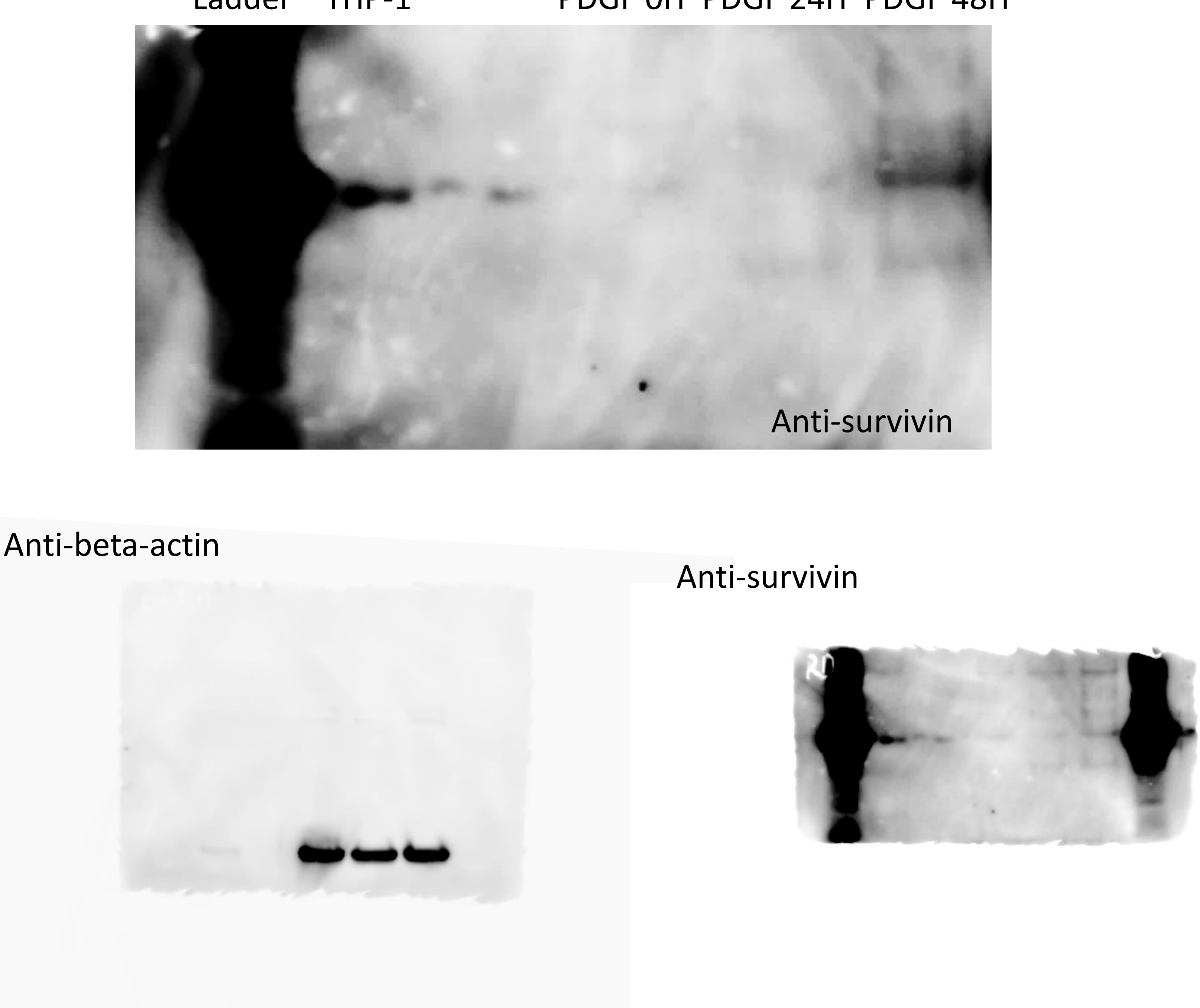
Ladder
THP-1
PDGF 0H
PDGF 24H
PDGF 48H
Anti-survivin
Anti-beta-actin
Anti-survivin

Figure 4c -1

Si RNA control | siRNA birc5 | Ladder

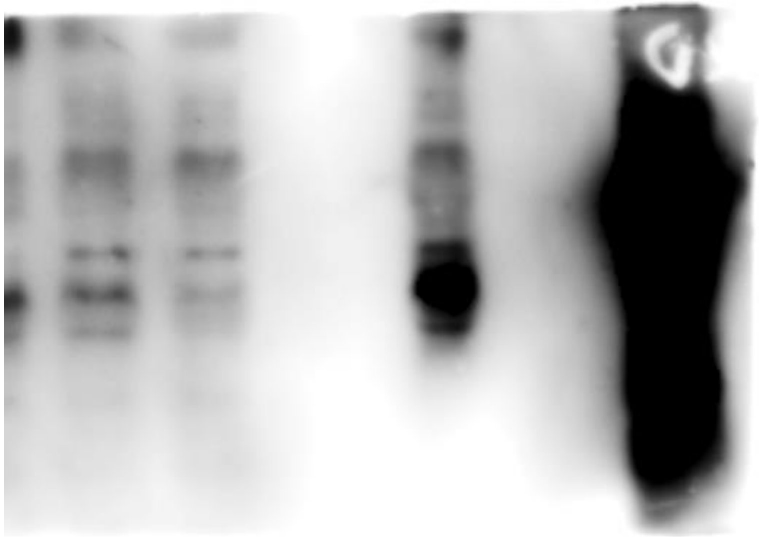

Anti-survivin

Si RNA control | siRNA 2B | Ladder

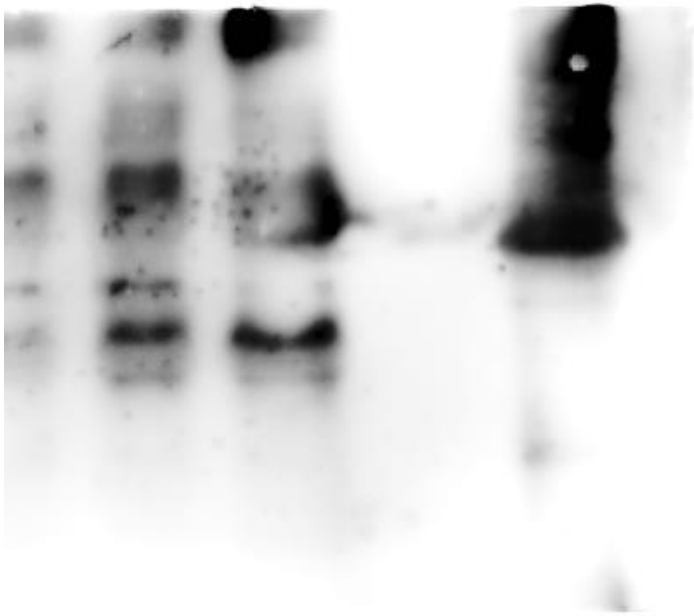

Anti-survivin

* When the image of Figure 5c was taken, left side of membrane was out of frame.

Figure 4c -2

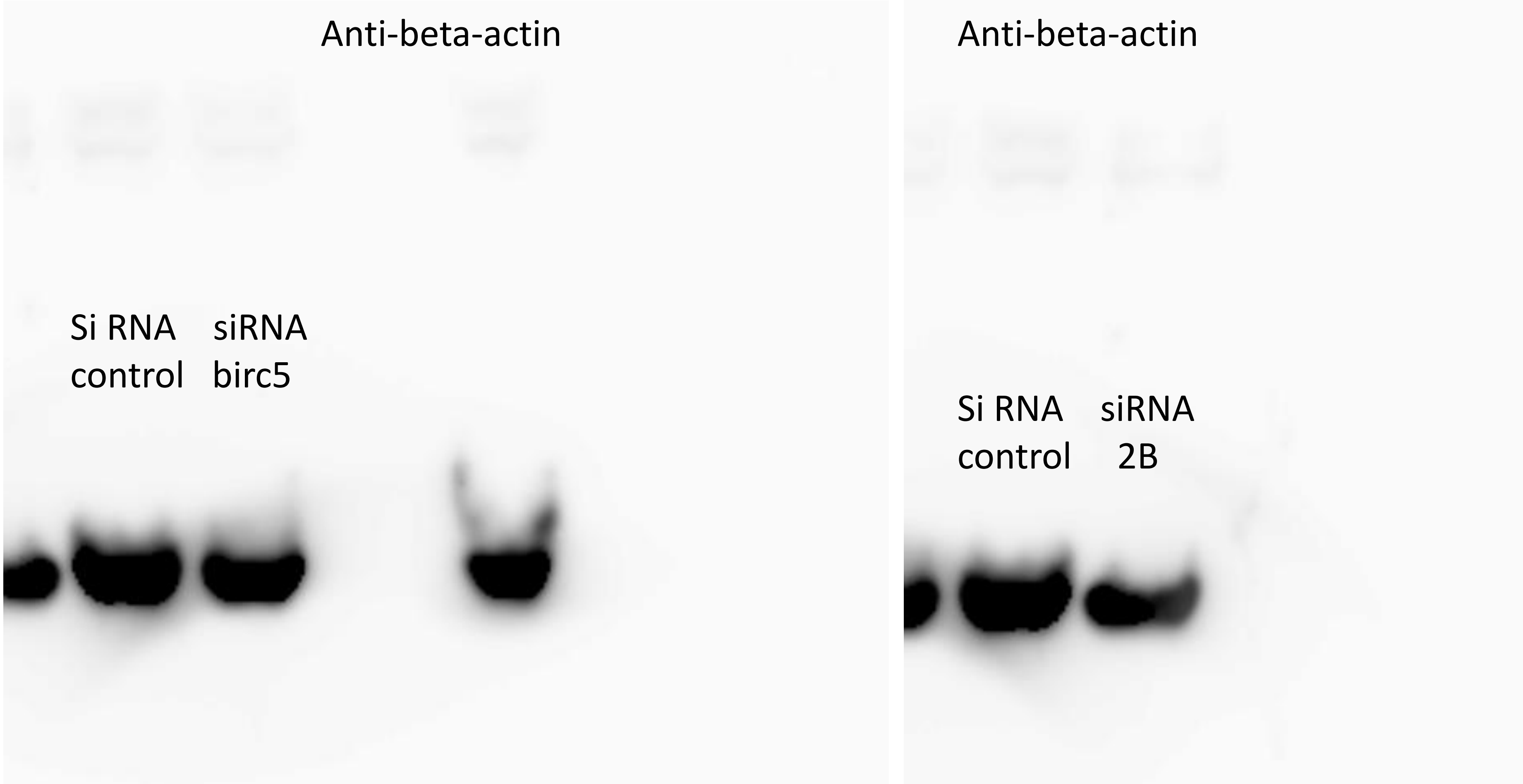

* When the image of Figure 5c was taken, left side of membrane was out of frame.

Figure 5a

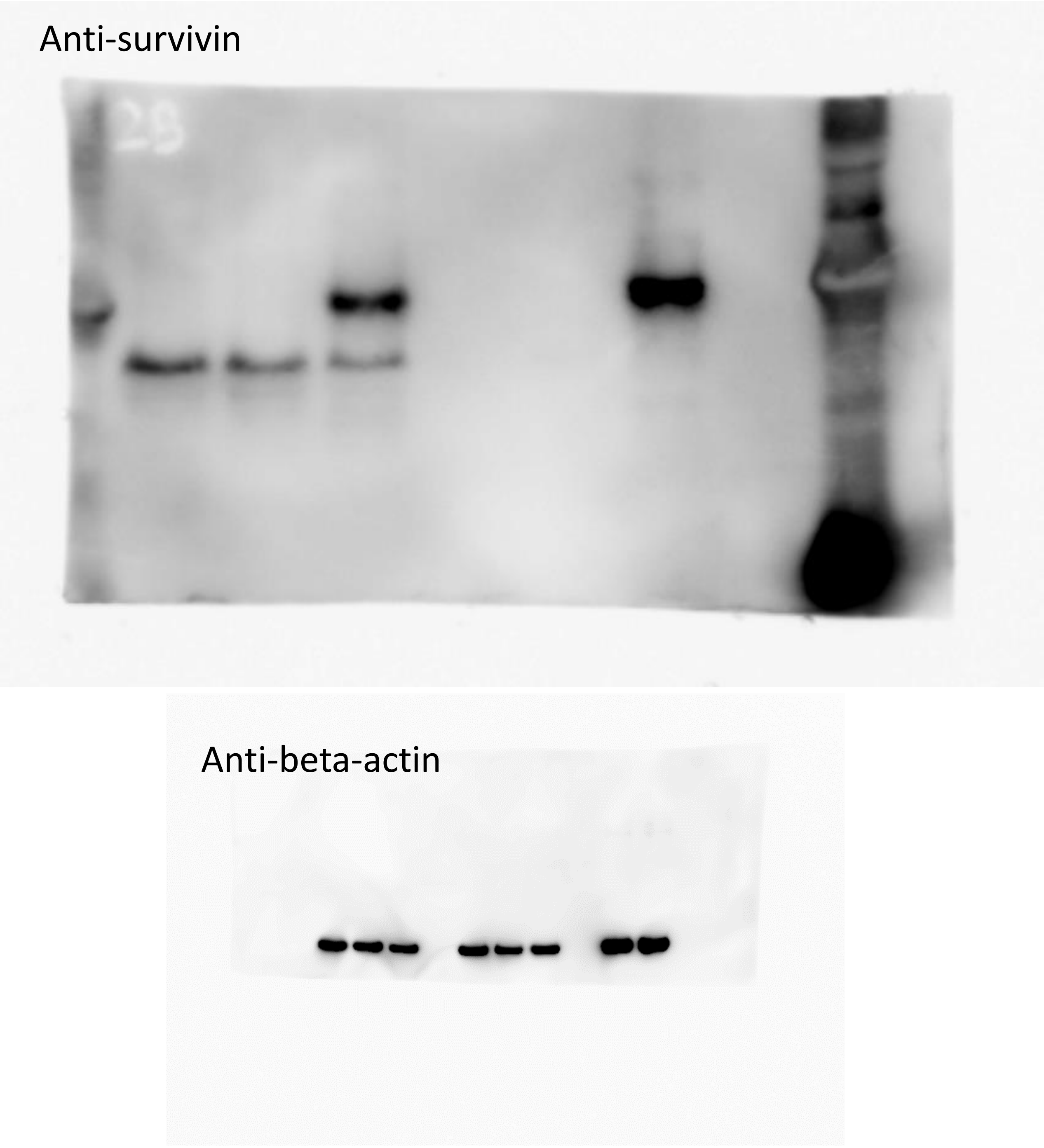

Supplemental Table 1. Patients' background regarding the serum survivin values using ELISA

	RA (n=22)	Controls (n=16)	p-value
Age	58.8 ± 15.6	53.9 ± 19.8	0.404 [a]
Sex (Female / Male)	F 17 / M 5	F 14 / M 2	0.422 [b]

Controls consisted of osteoarthritis patients and healthy individuals.

RA, rheumatoid arthritis.

Mean ± Standard Deviation (SD)

[a] t-test

[b] chi-square test

Supplemental Table 2. Patients' background regarding real-time PCR from whole synovial tissues

mean +- SD	RA (n=9)	OA (n=6)	p-value
Age	68.4 ± 7.2	74.1 ± 7.9	0.183 [a]
Sex (Female / Male)	F 9 / M 0	F 6 / M 0	1.000 [b]

RA, rheumatoid arthritis; OA, osteoarthritis.

Mean ± Standard Deviation (SD)

[a] t-test

[b] chi-square test

Supplemental Table 3. Patients' background regarding the serum survivin-2B values using ELISA

mean +- SD	RA (n=35)	Controls (n=21)	p-value
Age	64.7 ± 11.4	59.6 ± 18.0	0.250 [a]
Sex (Female / Male)	F 32 / M 3	F 19 / M 2	0.733 [b]

Controls consisted of osteoarthritis patients and healthy individuals.

RA, rheumatoid arthritis.

Mean ± Standard Deviation (SD)

[a] t-test

[b] chi-square test

Supplemental Figure 1. The sorting of synovial tissues using FACS

Synovial cells were sorted using FACS.
After sorting, samlples were analyzed with real-time PCR.
The ratio of cell numbers of CD8+ T lymphocytes and CD14+ monocyte were low, therefore we were not able to measure the expression of survivin.

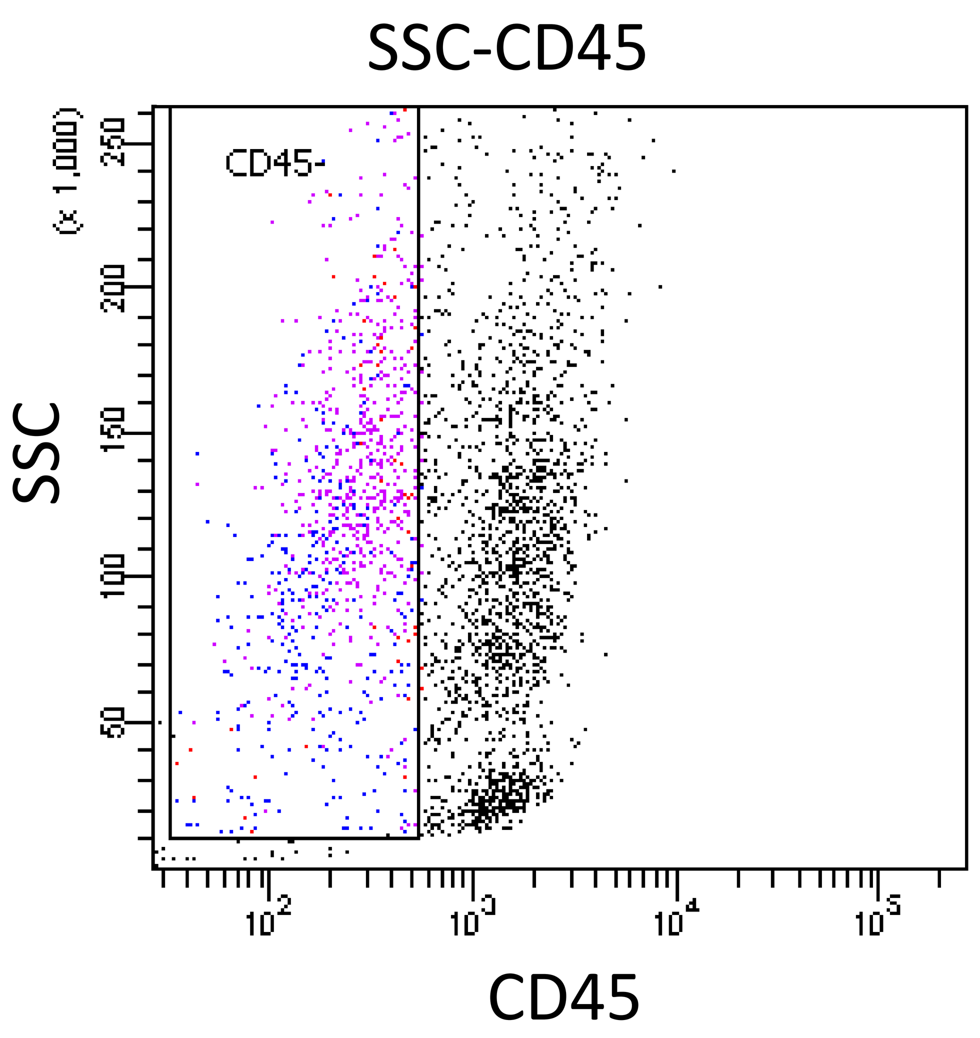

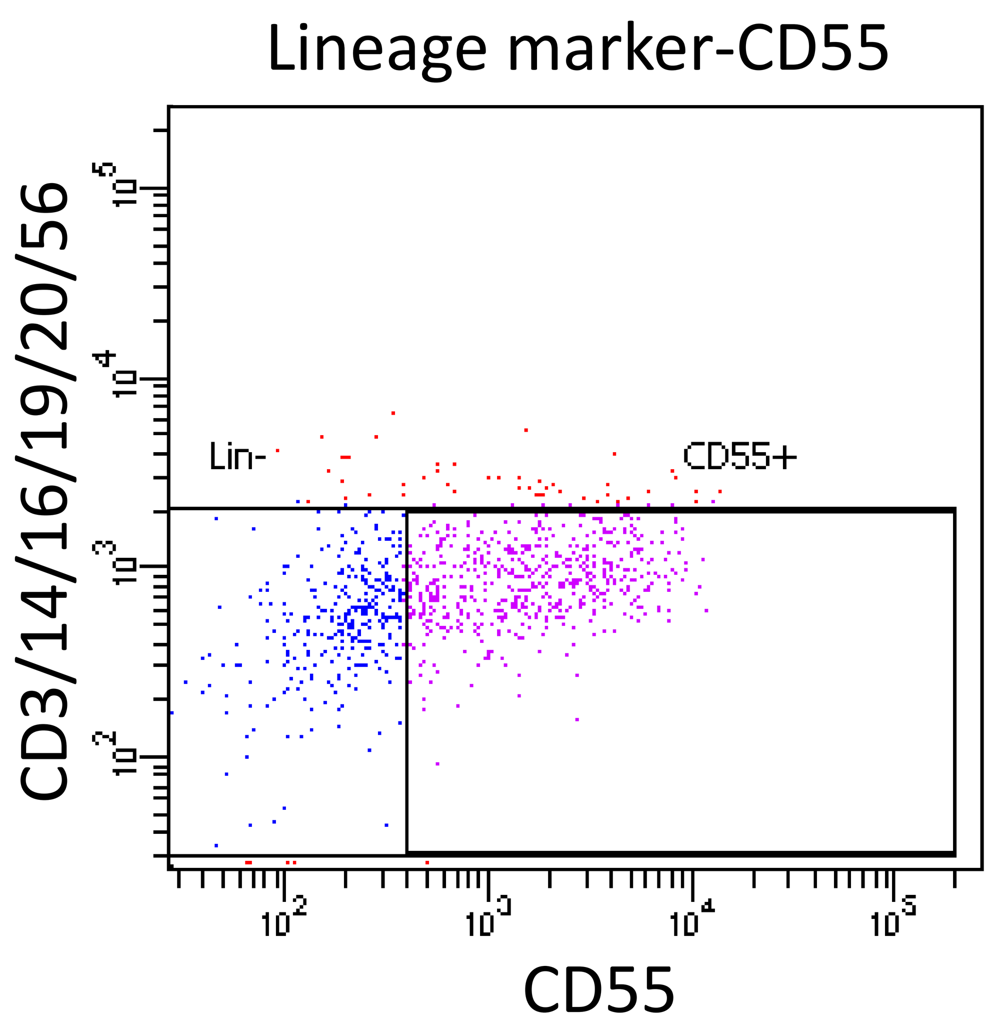

CD55+ FLS
(Lineage-CD45-CD55+)

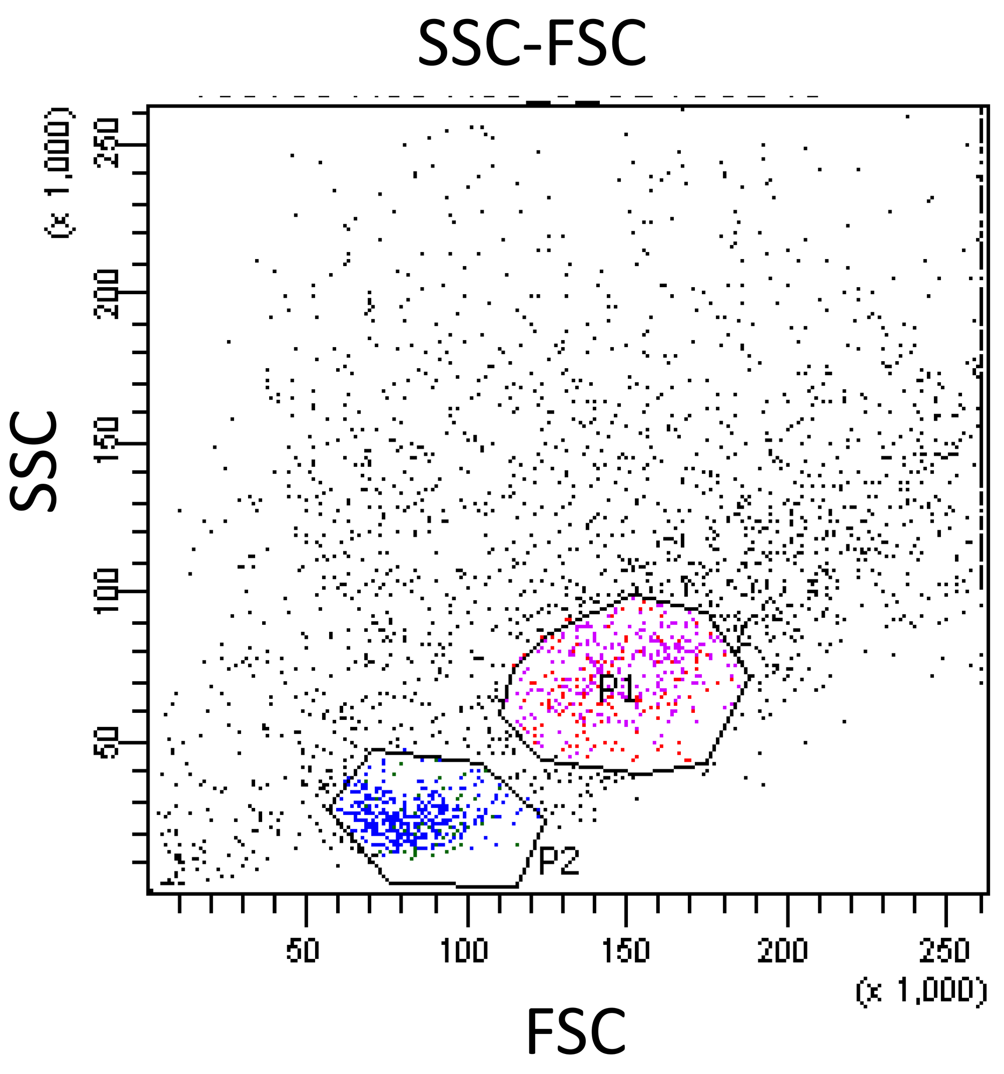

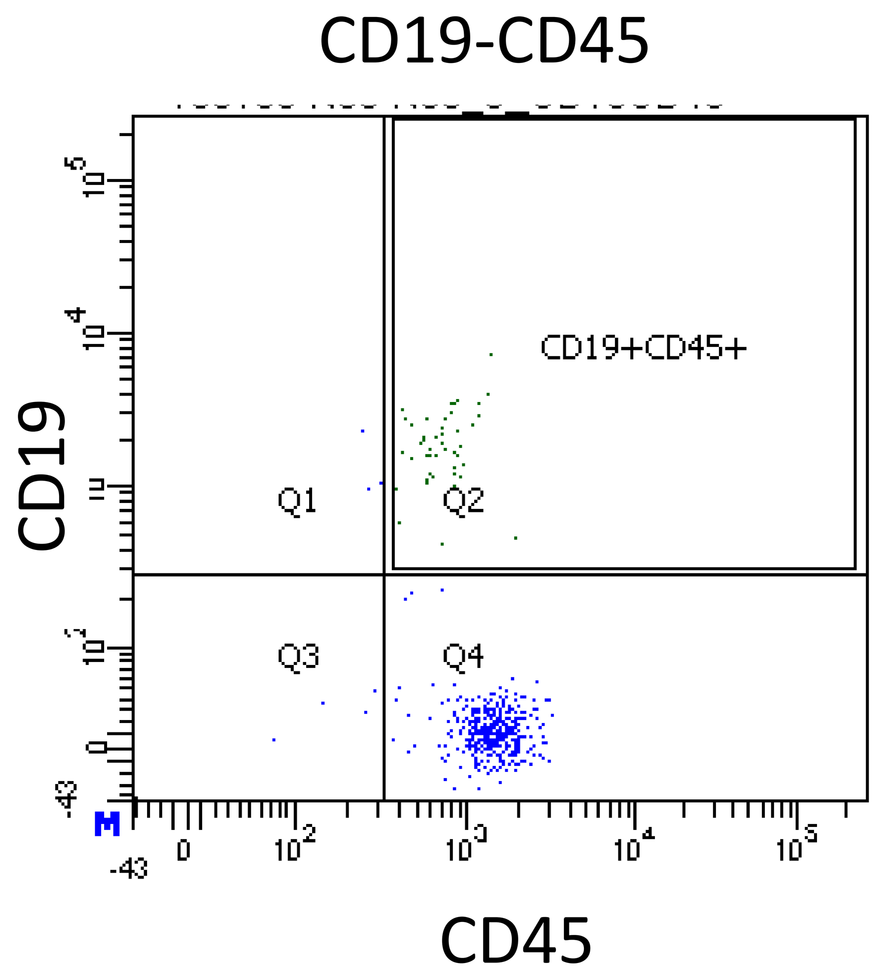

CD19+ B lymphocytes
(CD45+CD19+)

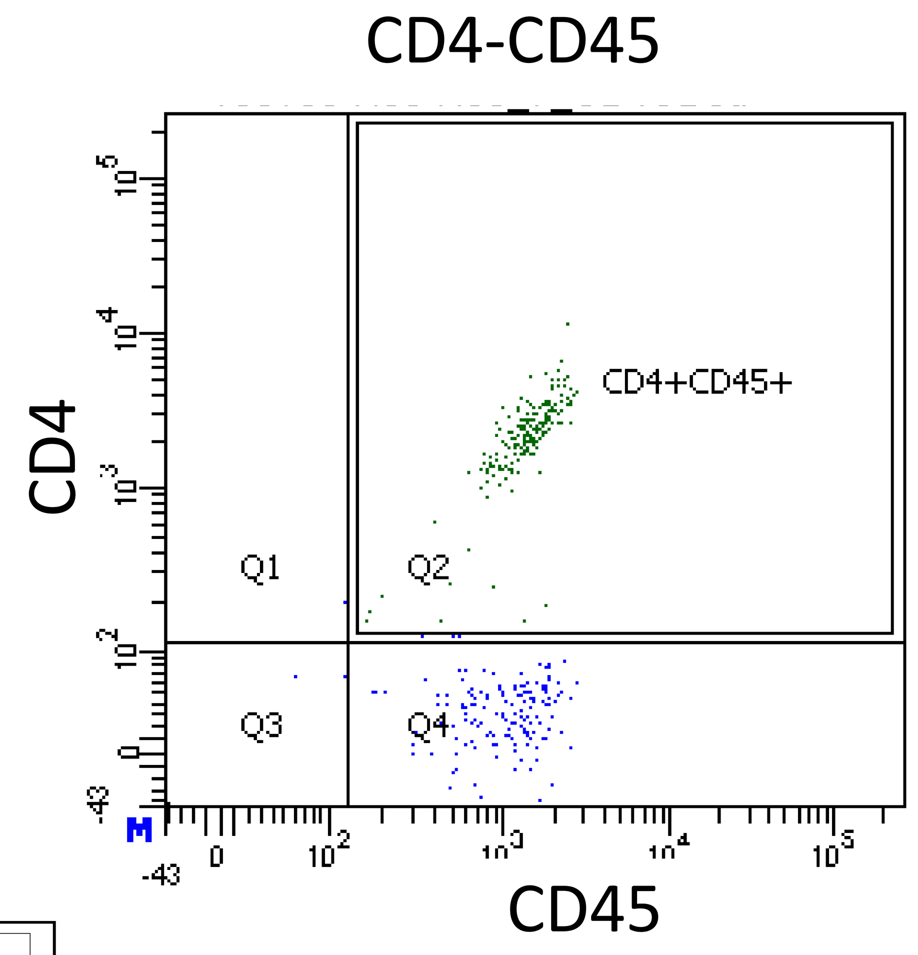

CD4+ T lymphocytes
(CD45+CD4+)

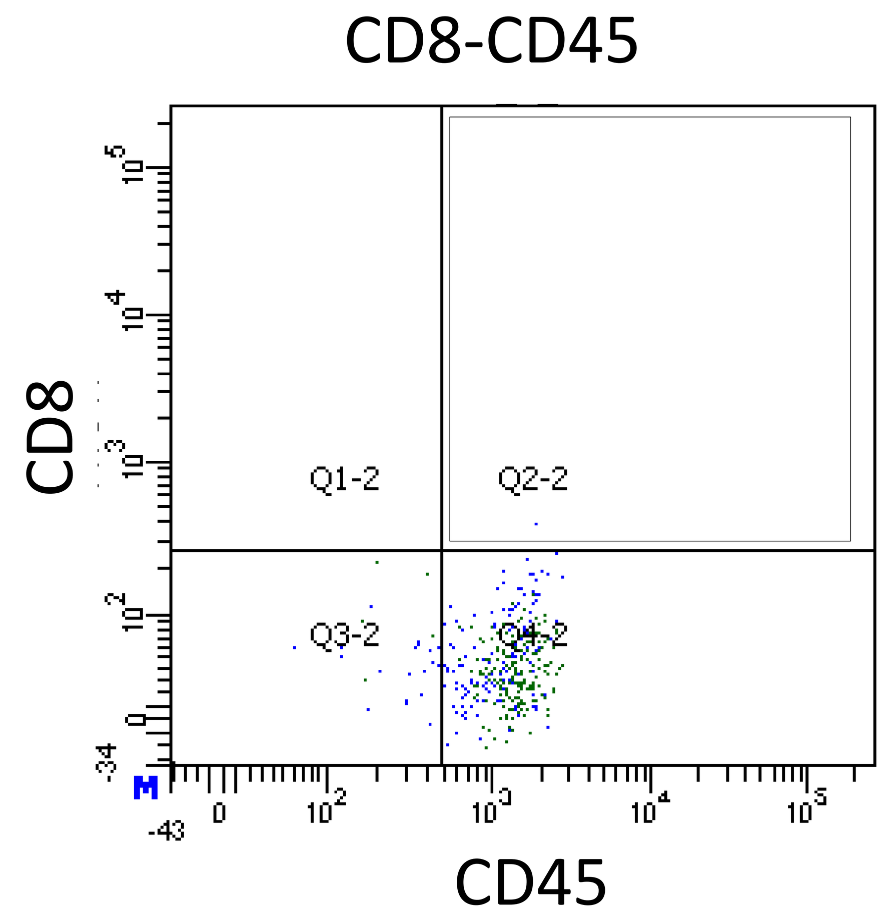

CD8+ T lymphocytes
(CD45+CD8+)

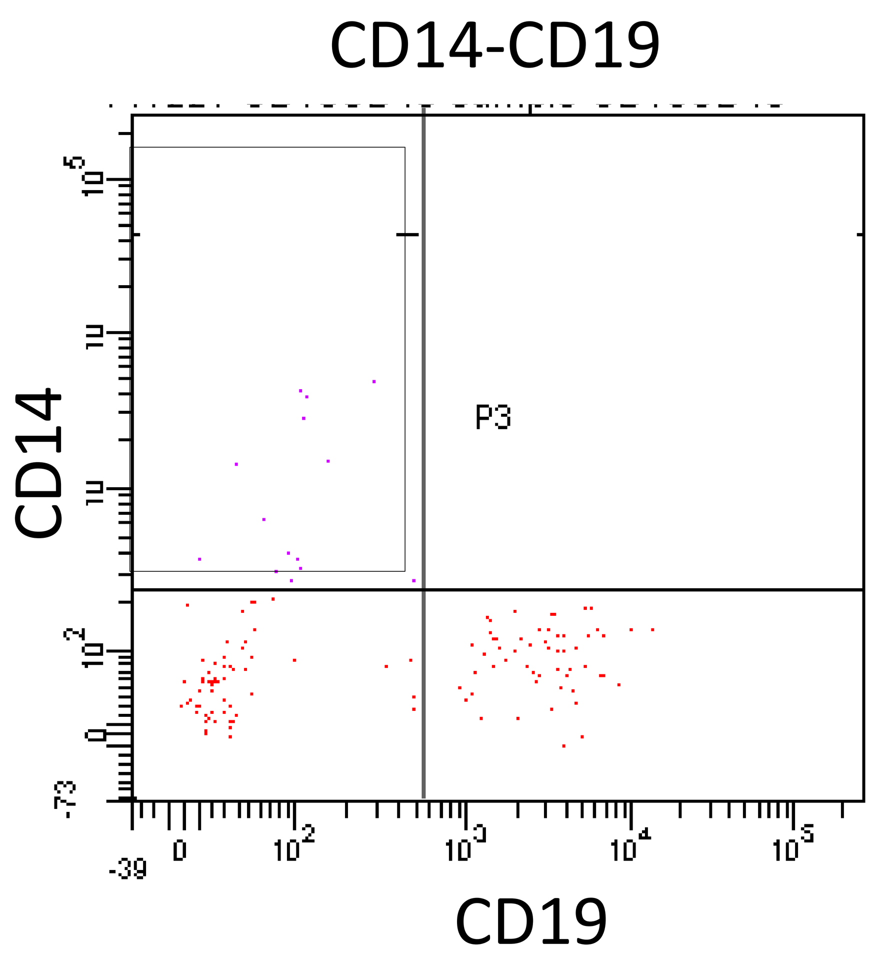

CD14+ monocytes
(CD14+)

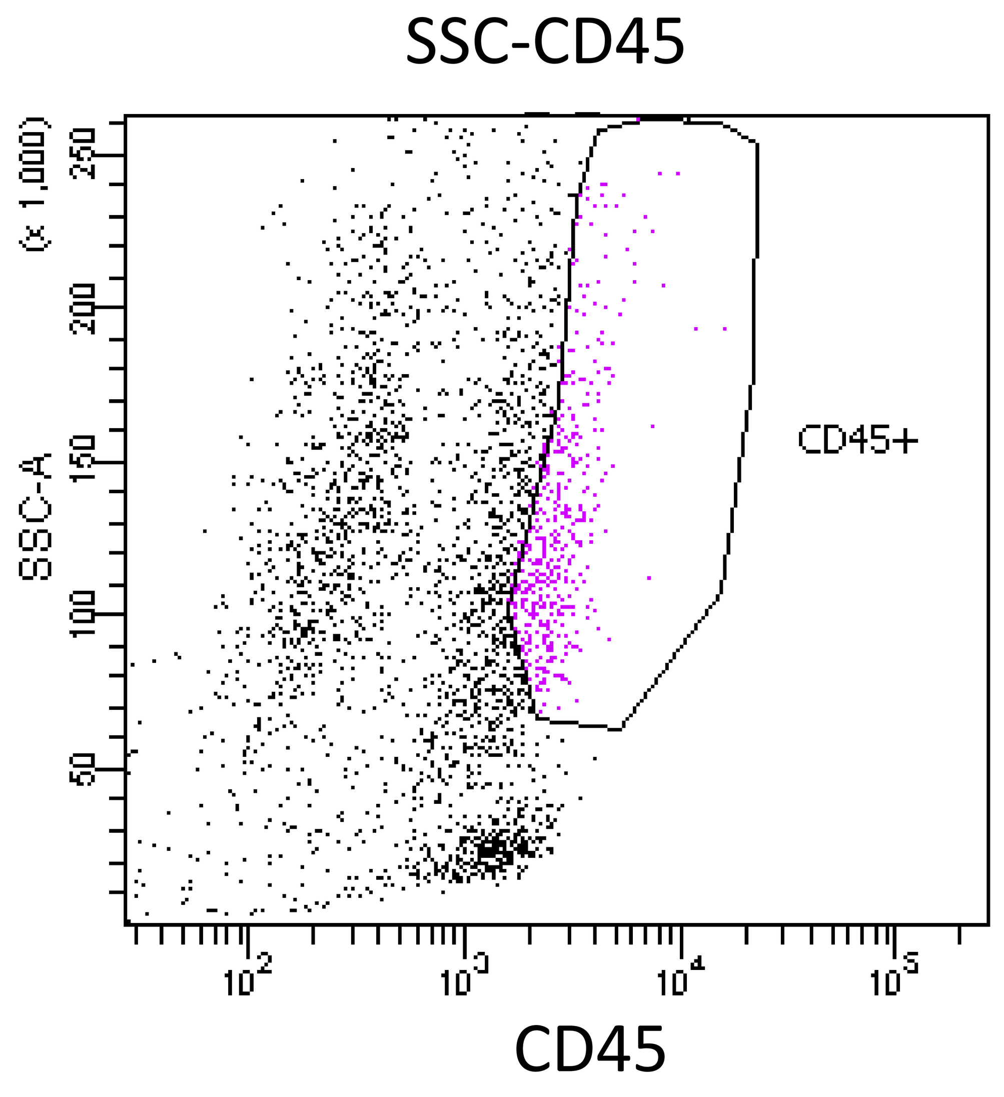

CD45+ neutrophil
(CD45+)
